# The complete chloroplast genome sequence of *Styrax serrulatus* Roxburgh (Styracaceae)

**DOI:** 10.1080/23802359.2021.1987170

**Published:** 2021-10-07

**Authors:** Lu Tian, Xiaogang Xu, Lili Tong, Chongli Xia, Yao Cheng

**Affiliations:** a Co-Innovation Center for Sustainable Forestry in Southern China, College of Biology and the Environment, Key Laboratory of State Forestry and Grassland Administration on Subtropical Forest Biodiversity Conservation, Nanjing Forestry University, Nanjing, China; b State Environmental Protection Scientific Observation and Research Station for Ecology and Environment of Wuyi Mountains, Nanjing, China; c School of Horticulture & Landscape Architecture, Jinling Institute of Technology, Nanjing, China

**Keywords:** *Styrax serrulatus*, phylogenomics, Styracaceae, complete chloroplast genome

## Abstract

*Styrax serrulatus* Roxburgh (William Roxburgh 1832), which plays an important role in ecology and economy, is a deciduous species of Styracaceae. In this paper, we sequenced, assembled, and annotated the chloroplast (cp) genome of *S*. *serrulatus* by using the sequencing data from Illumina Novaseq platform (Illumina, San Diego, CA). The complete cp genome of *S*. *serrulatus* is 157,929 base pairs (bp) in length, containing a pair of inverted repeat regions (IRs) of 26,048 bp each, a large single-copy (LSC) region of 87,552 bp, and a small single-copy (SSC) region of 18,281 bp. It contains 133 genes, including 8 rRNA genes, 37 tRNA genes, 87 protein-coding genes, and 1 pseudo gene. The GC content of *S*. *serrulatus* cp genome is 36.96%. The phylogenetic analysis suggests that *S*. *serrulatus* is a sister species to *Styrax agrestis* in Styracaceae.

*Styrax serrulatus* Roxburgh is a prominent species of Styracaceae, around 4–12 m tall, and 9–25 cm diameter of trunk at breast height, which mainly distribute in sparse forests at an altitude of 500–1700 m in the south and southwest (S Guangdong, S Guangxi, Hainan, Taiwan, SE Xizang and S Yunnan) of China and in the Bhutan, India, Laos, W Malaysia, Myanmar, Nepal, Thailand and Vietnam (Huang and Grimes 2003). It possesses high value for ornamental, timber, and medicinal purposes. However, there has been little progress on its complete chloroplast genome. In this work, we characterized the complete cp genome sequence of *S. serrulatus* (GeneBank accession number: MZ152917) based on Illumina pair-end sequencing data to provide a valuable complete cp genomic resource.

The fresh leaves of *S. serrulatus* were collected from Xishuangbanna Tropical Botanical Garden (**latitude** 21.6833 and **longitude** 101.4667) in Jinghong, Yunnan, China. A specimen was deposited at the herbarium of Nanjing Forestry University (contact person: xuehongma@njfu.edu.cn) under the voucher number NF2021019.

By using ultrasound to break DNA, the fragments of DNA were passivated, repaired and bonded. After the genomic DNA of the sample was qualified, the DNA was fragmented by a mechanical interruption method (ultrasound), and then the fragmented DNA was separately purified, while the end repaired, A was added at 3′-end, and the sequencing adapter was connected. The DNA fragments were selected by agarose gel electrophoresis. The sample of genome sequencing library was formed by PCR amplification, which was carried out on Illumina Novaseq 6000 system (Illumina, San Diego, CA) with PE150 reads by Nanjing Genepioneer Biotechnologies Inc. (Nanjing, China).

The original reads was filtered by fastp (version 0.20.0), and the clean data were assembled into chloroplast genome with SPAdes (Bankevich et al. 2012). Then, the reference sequence (Genebank accession number: NC041127.1) was used for quality control after assembly. The assembled genome was annotated using CpGAVAS (Liu et al. 2012). To examine the phylogenetic position of *S. serrulatus*, a multiple sequence alignment (MSA) analyses was performed using MAFFT (Rozewicki et al. 2019). Finally, a maximum-likelihood (ML) tree was deduced by IQ-Tree (Gao et al. 2018).

The complete chloroplast genome sequence of *S. serrulatus* was 157,929 bp in length, and contained a pair of inverted repeat (IRa and IRb) regions of 26,048 bp, which were separated by a large single copy (LSC) region of 87,552 bp, and a small single-copy (SSC) region of 18,281 bp. A total of 133 genes were encoded, including 87 protein-coding genes (80 CDS species), 37 tRNA genes (30 tRNA species), 8 rRNA genes (4 rRNA species), and 1 pseudo gene. Most of the genes occurred in a single copy, however, 7 protein-coding genes (*ndhB, rpl2, rpl23, rps12, rps7, ycf15*, and *ycf2*), 7 tRNA genes (*trnA-UGC, trnI-CAU, trnI-GAU, trnL-CAA, trnN-GUU, trnR-ACG*, and *trnV-GAC*), and 4 distinct rRNA genes (*4.5S, 5S, 16S*, and *23S*) were duplicated. A total of 10 protein-coding genes (*atpF, ndhA, ndhB, petB, petD, rpl16, rpl2, rpoC1, rps12*, and *rps16*) contained 1 intron while the other 2 genes (*clpP, ycf3*) had 2 introns each. The overall GC content of *S. serrulatus* genome was 36.96%, and the corresponding values in LSC, SSC, and IR regions were 34.80%, 30.28%, and 42.92%, respectively.

To reveal the phylogenetic evolution of *S. serrulatus*, we constructed a ML phylogenetic tree based on 40 cp genomes from Styracaceae and 5 cp genomes as outgroups from 3 taxa (Ebenaceae, Symplocaceae, Theaceae). We found that *S. serrulatus* was clustered with other families of Styracaceae with 100% bootstrap values (Figure 1). In addition, *S. serrulatus* was highly supported to be a sister species to *Styrax agrestis* in Styracaceae.

**Figure 1.**
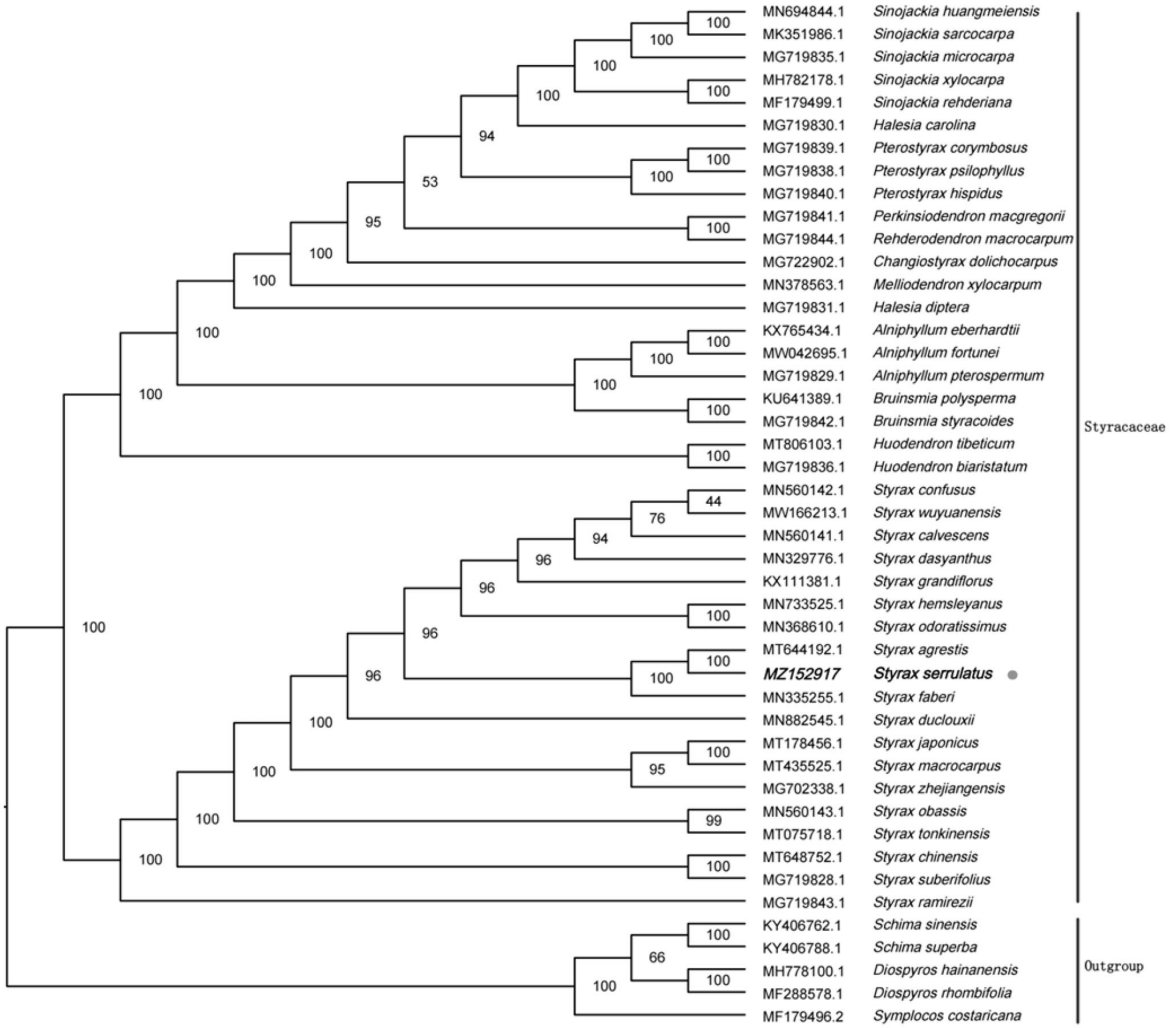
Maximum-likelihood tree showing the relationship among *Styrax serrulatus* and representative species within Styracaceae, based on whole chloroplast genome sequences, with 3 taxa from Ericales as an outgroup. The bootstrap supports the values shown at the branches.

## Data Availability

The genome sequence data that support the findings of this study are openly available in GenBank of NCBI at https://www.ncbi.nlm.nih.gov under the accession no. MZ152917. The associated BioProject, SRA, and Bio-Sample numbers are PRJNA739084, SRR14861495, and SAMN19771193, respectively.

## References

[CIT0001] Bankevich A, Nurk S, Antipov D, Gurevich AA, Dvorkin M, Kulikov AS, Lesin VM, Nikolenko SI, Pham S, Prjibelski AD, et al. 2012. SPAdes: a new genome assembly algorithm and its applications to single-cell sequencing. J Comput Biol. 19(5):455–477. 22506599 10.1089/cmb.2012.0021 PMC3342519

[CIT0002] Gao F-L, Shen J-G, Liao F-R, Cai W, Lin S-Q, Yang H-K, Chen S-L. 2018. The first complete genome sequence of *Narcissus latent* virus from *Narcissus*. Arch Virol. 163(5):1383–1386. 29392500 10.1007/s00705-018-3741-x

[CIT0003] Huang S-M, Grimes JW. 2003. Styracaceae. In: Wu Z-Y, Raven PH, Hong D-Y, editors. Flora of China, vols. 15 (Styracaceae). Beijing: Science Press; St. Louis: Missouri Botanic Garden Press; p. 260.

[CIT0004] Liu C, Shi L-C, Zhu Y-J, Chen H-M, Zhang J-H, Lin X-H, Guan X-J. 2012. CpGAVAS, an integrated web server for the annotation, visualization, analysis, and GenBank submission of completely sequenced chloroplast genome sequences. BMC Genomics. 13(715):715. 23256920 10.1186/1471-2164-13-715 PMC3543216

[CIT0005] Rozewicki J, Li S, Amada KM, Standley DM, Katoh K. 2019. MAFFT-DASH: integrated protein sequence and structural alignment. Nucleic Acids Res. 47(W1):W5–W10. 31062021 10.1093/nar/gkz342 PMC6602451

[CIT0006] William R. 1832. Descriptions of Indian Plants (Flora Indica). 2:415–416.

